# Supplementary material for: Diverse Terpenoids and Their Associated Antifungal Properties from Roots of Different Cultivars of Chrysanthemum Morifolium Ramat
Source: Molecules. 2020 Apr 29;25(9):2083. doi: 10.3390/molecules25092083 (PMC7248984; doi:10.3390/molecules25092083)
Supplement: Supplementary file 1 [file molecules-25-02083-s001.pdf]

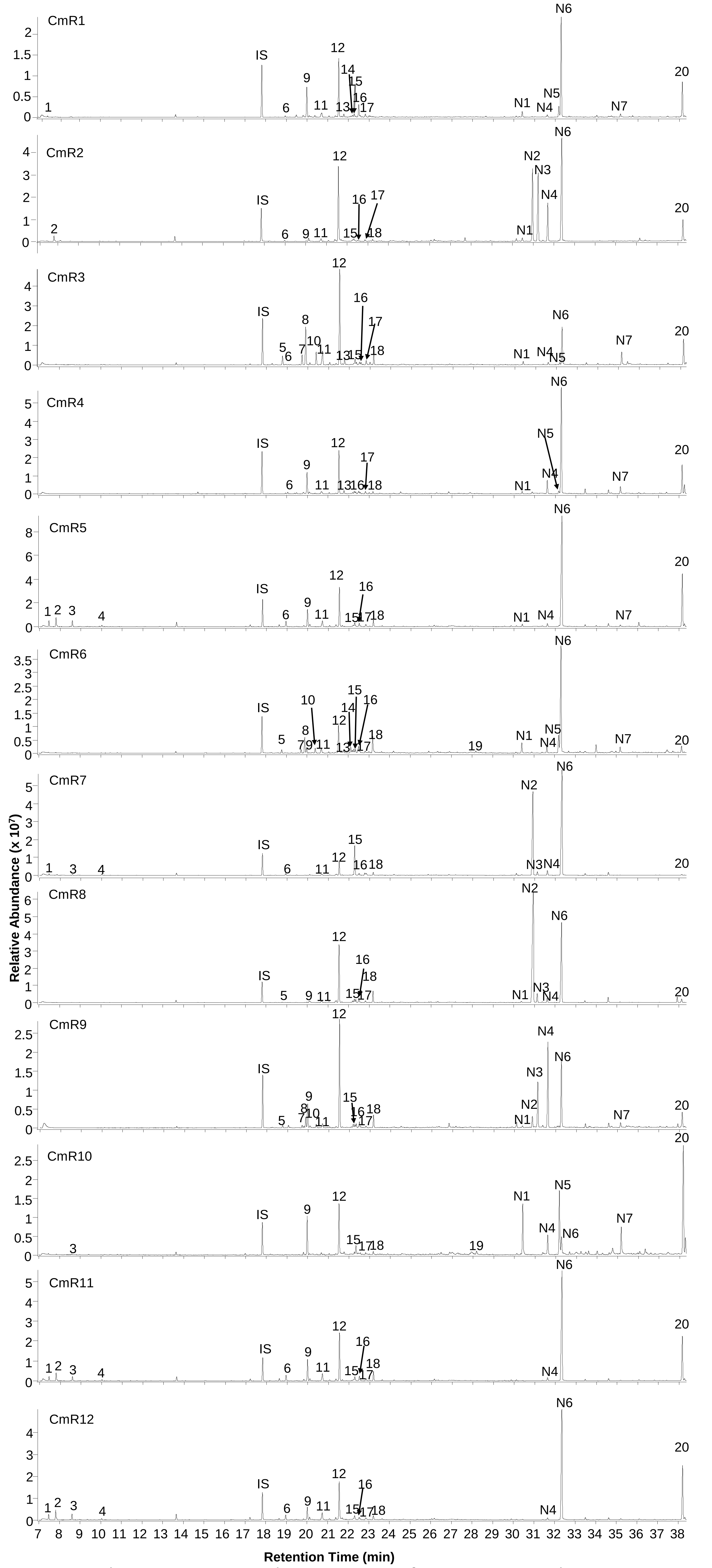

Figure S1 The representative Chromatogram of CmR1-CmR12. The major terpenoids were labeled with 1-20. 1. α-pinene,2.α-fenchene,3.β-pinene,4.para-cymene,5.silphinene, 6. α-longipinene,7.modephene, 8.α-isocomene, 9.β-elemene, 10.β-isocomene, 11.(E)-β-caryophyllene, 12.(E)-β-farnesene, 13.β-copaene, 14.δ-bisabolene, 15.neollocimene, 16.α-zingberene, 17.β-bisabolene, 18.β-sesquiphellandrene, 19.γ-Costol, 20.unidentified diterpene. N1-N7 represent the non-terpenoid compounds. N1. 3a,9b-Dimethyl-1,2,3a,4,5,9b-hexahydrocyclopenta[a]naphthalen-3-one, N2. 1,6-Dioxaspiro[4.4]non-3-ene, 2-(2,4-hexadiynylidene)- N3. (Z)-2-(Hexa-2,4-diyn-1-ylidene)-1,6-dioxaspiro[4.4]non-3-ene, N4. Azulen-2-ol, 1,4-dimethyl-7-(1-methylethyl)-, N5. Lovastatin, N6. (E)-2-(Hepta-2,4-diyn-1-ylidene)-1,6-dioxaspiro[4.4]non-3-ene, N7. 1-(4-(Isobutyryloxy)-3-methoxyphenyl)allyl 2-methylbutanoate

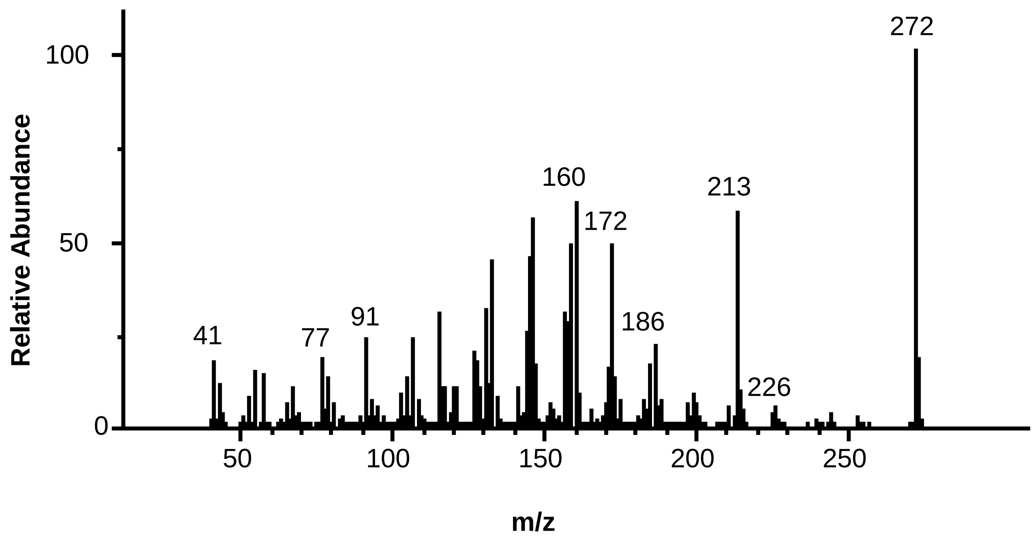

Figure S2 Mass spectrum of the unidentified diterpene from the root extract in CmR5.
